# An insight into pharmacokinetics and dose optimization of antimicrobials agents in elderly patients

**DOI:** 10.3389/fphar.2024.1396994

**Published:** 2024-09-30

**Authors:** Guanshuang Fu, Weijia Sun, Zhaoyi Tan, Beibei Liang, Yun Cai

**Affiliations:** ^1^ Centre of Medicine Clinical Research, Department of Pharmacy, PLA General Hospital, Beijing, China; ^2^ College of Pharmacy, Chongqing Medical University, Chongqing, China; ^3^ Medical Supplies Center of PLA General Hospital, Beijing, China

**Keywords:** elderly, pharmacokinetics, antimicrobial agents, drug resistance, anti-infective therapy

## Abstract

The global elderly population is on the rise, and infections tend to have a higher mortality rate among older individuals. Aging is associated with the progressive impairment of multi-organ function, which can impact the pharmacokinetics of antimicrobials, potentially leading to the failure of anti-infective therapy. With the increasing life expectancy, a significant growth in the elderly demographic, and the escalating costs of healthcare, gaining a thorough understanding of pharmacokinetic changes in the elderly holds crucial clinical significance. This review compiles findings from published studies, offering a comprehensive overview of the pharmacokinetics of various antimicrobials in both adults and the elderly. Furthermore, it delves into advancements in pharmacokinetic methods specific to the elderly population.

## 1 Introduction

The aging of the population, specifically those aged 65 and above, is an undeniable contemporary trend. Thanks to improvements in global living standards and enhanced medical care, the lifespan of the elderly is steadily increasing, resulting in a substantial growth in their numbers. According to the World Social Report 2023 (Leaving No One Behind In An Ageing World, 2023), the global population of elderly individuals has more than tripled, rising from approximately 260 million in 1980 to 761 million in 2021. Projections indicate that by 2030, this number is expected to surpass 1 billion and eventually exceed 1.6 billion by 2050. As we approach the end of the 21st century, the world could be home to nearly 2.5 billion elderly people.

Infection stands as a pivotal contributor to escalated disease progression and mortality among the elderly. Typically, the frequency and severity of infections escalate with advancing age (Bouza et al., 2020). Statistics from 2017 reveal that globally, the proportion of sepsis-related deaths, stemming from infectious diseases, is roughly 10% higher among individuals aged 65–89 than those aged 30–50 (Rudd et al., 2020). Moreover, mortality data concerning antimicrobial resistance (AMR) across countries in the WHO Region of the Americas for 2019 illustrate a steep surge in deaths attributed to AMR starting around age 65 (Antimicrobial Resistance Collaborators, 2023). Research underscores that within the initial 2 years of the COVID-19 pandemic, over 80% of global COVID-19 fatalities occur among individuals aged 60 and above (World Health Statistics, 2023). Furthermore, both the prevalence and fatality rates of lung infections are markedly elevated among older adults compared to their younger counterparts, with mortality from community-acquired pneumonia among older hospitalized patients more than doubling from 6% in those aged 65% to 15% in those over 90 (González-Castillo et al., 2014; Fernández-Sabé et al., 2003). Despite these glaring statistics, a significant treatment gap persists between the elderly and adults, as most drugs are developed with an adult population focus. In clinical practice, antimicrobials deemed effective and safe in adults may exhibit unexpectedly lower efficacy or provoke adverse reactions in elderly patients at standard recommended dosages. Such scenarios heighten the risk of inappropriate drug dosing among the elderly (Soraci et al., 2023).

As the elderly population continues to grow, the issue of drug safety and effectiveness in this demographic becomes increasingly prominent. The functions of many organs and systems undergo gradual degradation due to aging (Klotz, 2009). The aging nervous system, for instance, leads to an increased permeability of the blood-brain barrier, facilitating a greater influx of drugs from the plasma into the central system. Consequently, this amplifies drug distribution within the central system, elevating the incidence of adverse reactions in that area (Farrall and Wardlaw, 2009). Moreover, the significant degradation of kidney function in the aging body is noteworthy. This is characterized by reduced glomerular number, glomerular filtration rate, and renal blood flow. These factors impact the metabolism and excretion of drugs primarily processed by the kidneys (Thomson, 1995). Consequently, antimicrobial agents eliminated mainly by kidney tend to accumulate in the body, leading to adverse reactions. Additionally, elderly patients are often burdened with a variety of underlying conditions such as hypertension and diabetes. Consequently, the elderly constitute a widely medicated population, with each individual typically taking an average of 2–5 types of drugs daily (Kennerfalk et al., 2002). The combination of multiple drugs heightens the likelihood of interactions, presenting additional challenges for doctors when prescribing appropriate dosages of antimicrobials for the elderly (Gnjidic et al., 2018; Khan and Roberts, 2018).

Hence, providing a comprehensive delineation of the pharmacokinetic (PK) parameters of antimicrobials in the elderly population holds immense value in ensuring the delivery of safe and effective anti-infective therapy for elderly patients. This review endeavors to consolidate the understanding of the pharmacokinetics of diverse antimicrobial agents and highlight the advancements in research methodologies pertaining to pharmacokinetics in the elderly population.

## 2 ADME process in the elderly

The PK characteristics undergo significant alterations with age, particularly in the elderly population. These changes encompass a decline in renal filtration and excretion functions, gastrointestinal absorption function, and hepatic metabolic function (Koren et al., 2019). As a result, the processes associated with antimicrobial agents, including absorption, distribution, metabolism, and excretion (ADME), experience certain modifications in old age. In general, these changes tend to culminate in an elevated accumulation of drugs within the body.

### 2.1 Absorption

As age advances, the physiological characteristics of the gastrointestinal tract undergo gradual transformations. These changes predominantly involve a reduction in gastric acid secretion, gastrointestinal smooth muscle motor function, and gastrointestinal blood flow (Orr and Chen, 2002). Numerous studies indicate that, whether in a fasting or fed state, the pH value of the stomach increases, and gastric emptying slows in the elderly due to diminished gastric acid secretion and weakened motor function of the gastrointestinal smooth muscle (Vinarov et al., 2021; Giarratano et al., 2018). For instance, a higher gastric pH and reduced gastrointestinal peristalsis can diminish the solubility of posaconazole oral suspension and shorten its residence time in the stomach, consequently leading to a decrease in its plasma concentration (Chen et al., 2020). However, for certain oral antimicrobial agents like ciprofloxacin tablets, despite a decreased rate of drug absorption due to increased gastric pH in the elderly, the total amount of drug absorption remains unaffected owing to the prolonged residence time of drugs in the gastrointestinal tract (Lebel and Bergeron, 1987). Furthermore, the weakened stability of gastrointestinal flora in the elderly accelerates the decline of gastrointestinal function, thereby slowing down drug absorption (Tiihonen et al., 2010).

### 2.2 Distribution

The PK distribution process undergoes substantial changes in the elderly. In comparison to younger individuals, the elderly experience a notable decrease of 10%–15% in body water content and an increase of 20%–38% in adipose tissue (Beaufrère and Morio, 2000). Consequently, this results in a significant reduction in the distribution volume of hydrophilic antibacterial drugs such as aminoglycosides and penicillin, leading to a marked increase in drug peak concentration. It’s worth noting that in critically ill patients, the volume of distribution (V_d_) of hydrophilic drugs may increase due to continuous infusion and excessive capillary permeability (Udy et al., 2013). Conversely, the V_d_ of lipophilic drugs like tetracycline and amphotericin B increases, leading to a slower elimination (Shi and Klotz, 2011). Furthermore, as age progresses, the plasma protein binding rate of drugs in the body diminishes due to a reduction in plasma protein mass and quantity. Consequently, there is an increased risk of higher concentrations of free drug, a phenomenon more pronounced in individuals over the age of 70 (Celestin and Musteata, 2021).

### 2.3 Metabolism

The liver, a pivotal organ in the metabolism of most drugs, including antimicrobials, plays a crucial role in this process. In the elderly, there is a sharp decrease in the number of liver cells and local blood flow. Studies indicate that individuals at the age of 85 have approximately half the number of liver cells compared to their younger counterparts, coupled with a reduction of about 40 percent in local liver blood flow (McLean and Le Couteur, 2004). Consequently, for drugs with high hepatic clearance, the bioavailability may increase as the elderly experience diminished liver clearance of these drugs. However, the relationship between liver clearance and age for drugs with low liver clearance is a matter of controversy, warranting further investigation (McLachlan and Pont, 2012). Moreover, numerous drugs commonly prescribed for the elderly may interact with antimicrobial agents through hepatic microsomal enzymes (Benson, 2017). Take voriconazole, for instance, it serves as an inhibitor of CYP3A4 and CYP2C19 enzymes, influencing the metabolic rate of their substrates. Therefore, the concurrent use of drugs interacting with CYP3A4 substrates during voriconazole therapy may lead to increased drug concentration, heightening the risk of adverse reactions (Theuretzbacher et al., 2006).

### 2.4 Excretion

Most drugs undergo primary elimination through the kidneys, with some being excreted through bile, liver, the respiratory tract, and other pathways. In individuals over 65 years of age, the decline in kidney function exerts a more pronounced impact on pharmacokinetics than any other organ (Aymanns et al., 2010). As individuals age, the kidneys undergo a series of changes, including a decrease in the number of glomeruli and glomerular density, along with an increase in the incidence of glomerular sclerosis (Esposito et al., 2007). Furthermore, the length and volume of renal tubules significantly diminish, and the renal artery gradually hardens while renal blood flow gradually decreases (Bolignano et al., 2014). Beyond the age of 40, renal blood flow experiences a reduction of 10 percent per decade (Silva, 2005). Generally, in the elderly, decreased clearance, prolonged elimination half-life, and increased blood drug concentration of antibiotics eliminated by the kidneys are observed. These factors contribute to an extended retention time of the drug and elevate the likelihood of toxic accumulation in the body (Mühlberg and Platt, 1999).

## 3 Pharmacokinetic characteristics of antimicrobial agents in the elderly

In order to guarantee the safe and efficient utilization of anti-infective drugs among the elderly, comparative studies have been conducted for specific types of antimicrobials, encompassing both elderly and adult populations. Table 1 encapsulates the crucial PK parameters of anti-infective agents, as published to date, differentiating between older adults and adults. Particular emphasis should be directed towards antimicrobials exhibiting significant alterations in PK parameters within the elderly demographic.

**TABLE 1:** Comparison of pharmacokinetic parameters of some antimicrobial agents in adults and the elderly.

		Group	Study type	C_max_ (mg·L^−1^)	T_1/2_ (h)	V_d_ (L)	CL (L·h^−1^)	CL_r_ (mL·min^−1^)	AUC (h·mg·L^−1^)
Penicillins	Penicillin (Yaoguo et al., 2001) (Dosage 0.96 g)	Adults (27.8 ± 5.0)	PK STUDY	50.7 ± 10.6	0.77 ± 0.1	37.8 ± 13.3		429.4 ± 100.3	31.4 ± 4.5
		Elderly (69.8 ± 5.4)	PK STUDY	51.4 ± 9.8	1.22 ± 0.4	40.1 ± 8.3		257.3 ± 56.2	43.5 ± 6.2
	Piperacillin (Yaoguo et al., 2001) (Dosage 1 g)	Adults (27.8 ± 5.0)	PK STUDY	58.0 ± 13.5	1.1 ± 0.1	20.53 ± 6.1		145.9 ± 27.9	79.4 ± 13.9
		Elderly (69.8 ± 5.4)	PK STUDY	70.8 ± 7.5	1.7 ± 1.1	26.47 ± 14.7		105.2 ± 19.3	94.2 ± 12.4
	Ampicillin /Sulbactam (Meyers et al., 1991) (Dosage 2 g/1 g)	Adults (20–40)	PK STUDY	99.78 ± 26.94/52.21 ± 14.76	0.86 ± 0.15/0.93 ± 0.15	31.4 ± 13.12/24.98 ± 4.66	17.35 ± 3.03/15.30 ± 3.18	172.52 ± 61.55/10.61 ± 4.00	118.44 ± 20.99/67.95 ± 14.41
		Elderly (65–85)	PK STUDY	112.40 ± 34.30/59.10 ± 19.90	1.35 ± 0.29/1.58 ± 0.29	26.33 ± 8.75/23.54 ± 7.71	11.88 ± 3.34/9.76 ± 2.77	71.54 ± 28.30/3.96 ± 1.47	182.15 ± 57.79/110.37 ± 32.70
	Amoxicillin /Clavulanate	Adults (Lee et al., 2017) (29.9 ± 5.3) (Dosage 750 mg/375 mg)	PK STUDY	4.12 ± 1.05/1.37 ± 0.60	1.43 ± 0.25/1.14 ± 0.11	86.48 ± 23.96/84.36 ± 39.39	41.92 ± 10.03/51.55 ± 24.62		18,751 ± 3,903/4,357 ± 1797
		Elderly (Lee et al., 2020) (65–85) (Dosage 750 mg/187.5 mg)	PK STUDY	7.97 ± 2.77/2.61 ± 1.76	1.75 ± 0.22/1.30 ± 0.15	52.72 ± 11.22/80.42 ± 89.86	21.14 ± 5.01/45.00 ± 57.69		37,542 ± 9,523/8,474 ± 5,866
Cephalosporins	Ceftazidime /Avibactam (Li et al., 2019a)	Adults (18–65)	PPK	70.0/12.5					800/131
		Elderly (65–75)	PPK	77.1/13.2					997/156
	Cefazolin (Yaoguo et al., 2001) (Dosage 1 g)	Adults (27.8 ± 5.0)	PK STUDY	158.5 ± 18.9	1.9 ± 0.3	8.57 ± 1.3		44.5 ± 11.0	339.56 ± 79.9
		Elderly (69.8 ± 5.4)	PK STUDY	157.7 ± 22.8	2.5 ± 0.4	10.64 ± 1.8		34.7 ± 7.1	380.71 ± 61.4
	Cefperazon (Mingji et al., 2007) (Dosage 1 mg)	Adults Male (18–27)	PK STUDY	88.33 ± 12.93	1.55 ± 0.75	10.99 ± 0.62	5.52 ± 1.73		200.4 ± 82.9
		Elderly Male (65–74)	PK STUDY	102.03 ± 15.26	2.17 ± 0.57	12.76 ± 3.80	4.21 ± 1.23		256.3 ± 72.0
	Cefepime (Barbhaiya et al., 1992) (Dosage 1 mg)	Adults Male ±6 (30)	PK STUDY	75.1 ± 9.7	2.26 ± 0.51				149 ± 21
		Elderly Male ±2 (67)	PK STUDY	74.4 ± 11.9	3.05 ± 0.50				199 ± 30
Carbapenems	Ertapenem (Musson et al., 2004) (Dosage 1 g)	Adults (32.8 ± 6.0)	PK STUDY		3.8 ± 0.5			12.9 ± 4.3	572.1 ± 68.6
		Elderly (≥65)	PK STUDY		5.2			8.6 ± 2.2	781.5 ± 95.9
	Meropenem (Ljungberg and Nilsson-Ehle, 1992) (Dosage 500 mg)	Adults (20–34)	PK STUDY	35.6 ± 5.2	0.81			137 ± 25.1	39.6 ± 6.8
		Elderly (67–80)	PK STUDY	37.0 ± 6.2	1.27			99 ± 13.3	58.3 ± 10.0
	Biapenem (Dosage 300 mg)	Healthy Adults Subject (18–40) (Liu et al., 2013)	PK STUDY	18.50 ± 1.16			11.06 ± 1.16		
		Elderly Patients (≥65) (Namkoong et al., 2014)	PK STUDY	22.88			6.59		
	Doripenem (Dosage 500 mg)	Adults (22–30) (Kim et al., 2018)	PK STUDY	9.72	1.01				12.0
		Elderly (≥65) (Harada et al., 2013)	PK STUDY	20.0	1.20				31.6
Monobactams β-lactams	Aztreonam (Meyers et al., 1993) (Dosage 2 g)	Adults (18–30)	PK STUDY	160 ± 47.64	1.80 ± 0.51	17.23 ± 5.67	6.60 ± 1.58		294.42 ± 64.08
		Elderly (≥65)	PK STUDY	153.5 ± 36.31	3.10 ± 0.90	17.19 ± 2.14	4.32 ± 2.28		469.01 ± 144.03
Aminoglycoside antibiotics	Amikacin (Yaoguo et al., 2001) (Dosage 400 mg)	Adults (27.8 ± 5.0)	PK STUDY	43.6 ± 6.4	2.1 ± 0.2	16.5 ± 3.4		78.8 ± 10.6	76.5 ± 10.5
		Elderly (69.8 ± 5.4)	PK STUDY	40.7 ± 6.3	3.1 ± 0.4	18.4 ± 2.7		46.5 ± 10.7	99.9 ± 20.3
	Tobramycin (Champoux et al., 1996) (Dosage 80 mg)	Adults (29.4 ± 11.3)	PK STUDY	2.5 ± 0.6	2.73 ± 1.17	23.6 ± 4.8	6.9 ± 3.12		12.33 ± 3.75
		Elderly (65.8 ± 1.9)	PK STUDY	2.7 ± 0.8	3.53 ± 0.97	26.2 ± 8.2	5.28 ± 1.44		16.33 ± 4.8
	Gentamicin	Adults (Bos et al., 2019) (20–86)	PPK			20	5.5		
		Elderly (Johnston et al., 2014) (65–96)	PPK			14.9	2.96		
Macrolide antibiotics	Telithromycin Perret et al. (2002) (Dosage 800 mg)	Adults (18–40)	PK STUDY	1.64				190.3	8.40
		Elderly (65–85)	PK STUDY	1.91				141.7	10.83
	Dirithromycin Varanese (1993) (Dosage 500 mg)	Adults (18–50)	PK STUDY	0.36 ± 0.18					1.83 ± 1.09
		Elderly (≥65)	PK STUDY	0.85 ± 0.30					4.30 ± 3.10
	Clarithromycin Chu et al. (1992)	Adults (18–30)	PK STUDY	2.41 ± 0.67	4.9		28.56 ± 6.72	168 ± 35	18.87 ± 5.55
		Elderly (65–84)	PK STUDY	3.28 ± 0.80	7.7		18 ± 5.82	84 ± 31	30.76 ± 8.50
	Azithromycin Coates et al. (1991) (Dosage 500 mg)	Adults (22–39)	PK STUDY	0.41					2.5
		Elderly (67–80)	PK STUDY	0.38					3.0
Tetracycline antibiotics	Minocycline	Adults Male (JB, 1983) (Dosage 200 mg)	PK STUDY	3.5	15.7	115	3.36 ± 0.31		
		Debilitated Elderly Patients ±6(82 (Yamamoto et al., 1999) (Dosage 100 mg)	PK STUDY	7.22 ± 3.53	25.0 ± 16.4	32.9 ± 13.4	1.14 ± 0.49		
	Tigecycline (Muralidharan et al., 2005) (Dosage 100 mg)	Adults Male (18–50)	PK STUDY	0.861 ± 0.154	22.3 ± 15.3	554 ± 158	28.5 ± 11.8	48.3 ± 18.3	4.218 ± 2.033
		Elderly Male (>75)	PK STUDY	1.017 ± 0.112	19.0 ± 5.0	401 ± 58	18.7 ± 3.0	43.3 ± 10	5.472 ± 0.901
Quinolone antibiotics	Ofloxacin (Molinaro et al., 1992) (Dosage 300 mg)	Adults (24.6 ± 2.6)	PK STUDY	2.7 ± 0.7	6.2 ± 0.9		13.98 ± 1.998		21.5 ± 4.27
		Elderly (79–90)	PK STUDY	4.7 ± 0.9	8.5 ± 1.2		4.998 ± 0.996		83.3 ± 16.6
	Levofloxacin	Adults (Setiawan et al., 2022) (58.8 ± 16.4)	PPK			55.8 ± 35.3	1.12 ± 0.58		
		Elderly (Cojutti et al., 2017) (81.2 ± 7.8)	PPK			52.95 ± 21.57	2.53 ± 1.46		
	Sitafloxacin (Yaozh, 2023) (Dosage 100 mg)	Adults (25–35)	PK STUDY	0.91 ± 0.38	0.92 ± 0.20				4.86 ± 0.82
		Elderly (67–80)	PK STUDY	0.61 ± 0.23	3.80 ± 1.48				6.35 ± 1.51
	Ciprofloxacin Ljungberg and Nilsson-Ehle (1989) (Dosage 250 mg)	Adults (22–34)	PK STUDY	1.24 ± 0.32					4.7 ± 0.7
		Elderly (63–76)	PK STUDY	1.47 ± 0.4					6.4 ± 1.08
	Garenoxacin (Dosage 400 mg)	Adults (49.5 ± 17) Van Wart et al. (2004)	PPK				5.04 ± 1.32		85 ± 25
		Elderly (≥65) (Ohsaki et al., 2010)	PK STUDY				3.44		118
	Nemonoxacin (Dosage 500 mg)	Adults (25 ± 3) (Wu et al., 2015)	PK STUDY	10 ± 1.6	6.96 ± 1.42	86.67 ± 14.23			40.25 ± 8.59
		Elderly (54.30 ± 11.25) (Kang et al., 2019)	PK STUDY	6.870 ± 1.979	10.42 ± 1.22	156.77 ± 40.36			43.43 ± 9.28
	Delafloxacin (Hoover et al., 2016) (Dosage 250 mg)	Adults (18–40)	PK STUDY	3.58 ± 0.602	8.3	444 ± 605	19.3 ± 4.8		13.6 ± 2.85
		Elderly (≥65)	PK STUDY	4.62 ± 0.793	5.9	158 ± 147	13.7 ± 3.4		19.3 ± 4.84
	Lascufloxacin Totsuka et al. (2020) (Dosage 200 mg)	Adults (20–40)	PK STUDY	1.50 ± 0.247	15.6 ± 2.39	151 ± 22.1	6.73 ± 0.893	13.65 ± 3.55	30.2 ± 4.23
		Elderly (≥65)	PK STUDY	2.16 ± 0.438	16.6 ± 2.67	144 ± 25.4	6.06 ± 1.07	10.533 ± 1.62	33.8 ± 5.11
Oxazolidinones	Linezolid (Sisson et al., 2002) (Dosage 600 mg)	Adults Male (18–45)	PK STUDY	11.7 ± 2.3	5.3 ± 1.7			0.44 ± 0.07/kg	80.0 ± 18.0
		Elderly Male (≥65)	PK STUDY	11.9 ± 2.9	4.6 ± 1.3			0.31 ± 0.06/kg	74.3 ± 15.8
	Tedizolid (Flanagan et al., 2018) (Dosage 200 mg)	Adults (18–45)	PK STUDY	2.4 ± 0.5	11.8 ± 1.0	96.6 ± 21.0	5.7 ± 1.3		29.5 ± 5.7
		Elderly (≥65)	PK STUDY	2.6 ± 0.7	12.3 ± 1.3	91.6 ± 28.2	5.2 ± 1.6		34.2 ± 10.3
	Contezolid (Li et al., 2020) (Dosage 600 mg)	Healthy volunteers (18–32)	PPK	16.88	4.14	0.53/kg	0.16/kg		62.71
		Patients (18–70)	PPK	10.85	1.86	0.39/kg	0.19/kg		51.43
Polypeptide antibiotics	Polymyxin B	Adults (58.7 ± 15.1) Manchandani et al. (2018)	PPK			34.3 ± 16.4	2.5 ± 1.1		
		Elderly (≥65) (Wang et al., 2022a)	PPK			29.38 ± 10.82	1.98 ± 0.67		
	Norvancomycin (Zhang et al., 2008) (Dosage 1,000 mg)	Adults (18–65)	PPK		6.79 ± 5.18	40.07 ± 11.44	5.89 ± 2.08	118.15 ± 38.22	283.92 ± 161.66
		Elderly (≥65)	PPK		12.07 ± 12.19	52.67 ± 22.77	3.94 ± 1.73	71.44 ± 25.44	490.16 ± 564.36
	Vancomycin (Guay et al., 1993)	Adults (18–59)	PK STUDY		7.5 ± 6.7	67.0 ± 30.7			
		Elderly (60–92)	PK STUDY		17.8 ± 11.8	74.2 ± 32.3			
	Teicoplanin	Adults (21–38) (Thompson et al., 1992) (Dosage 6 mg/kg)	PK STUDY		138.0	1.0/kg	0.012/kg		
		Elderly (≥65) (Rosina et al., 1988) (Dosage 6 mg/kg)	PK STUDY		106.74 ± 5.03	1.63 ± 0.12/kg	0.011/kg		
	Telavancin	Adults (18–40) (Wong et al., 2008) (Dosage 7.5 mg/kg)/(Dosage 15 mg/kg)	PK STUDY	87.5 ± 12.8/186 ± 27	6.0 ± 0.6/7.5 ± 1.3	111 ± 32/kg/119 ± 14/kg	13.0 ± 3.6/kg/12.0 ± 1.9/kg		599 ± 96/1,282 ± 201
		Elderly (≥65) (Goldberg et al., 2010) (Dosage 10 mg/kg)	PK STUDY	87.7 ± 9.7	9.3 ± 1.3	156 ± 12/kg	12.2 ± 1.4/kg		673 ± 59
Antituberculosis agents	Isoniazid (Advenier et al., 1980) (Fast acetylators)	Adults (39 ± 15) (Dosage 4.9 ± 0.2 mg/kg)	PK STUDY			0.67 ± 0.32/kg	0.44 ± 0.12/kg		12.06 ± 3.31
		Elderly (89 ± 11) (Dosage 7.1 ± 0.5 mg/kg)	PK STUDY			0.54 ± 0.42/kg	0.50 ± 0.22/kg		16.18 ± 6.57
	Isoniazid (Advenier et al., 1980) (Slow acetylators)	Adults (36 ± 13) (Dosage 5.0 ± 0.8 mg/kg)	PK STUDY			0.68 ± 0.22/kg	0.22 ± 0.06/kg		24.45 ± 8.47
		Elderly (80 ± 10) (Dosage 6.7 ± 0.9 mg/kg)	PK STUDY			0.688 ± 0.463/kg	0.22 ± 0.11/kg		38.29 ± 18.74
	Rifampicin (Weng, 1990) (Dosage 450 mg)	Adults (35.9 ± 7.6)	PK STUDY	14.56 ± 6.45	2.39 ± 0.66	0.43 ± 0.11/kg	0.14 ± 0.03/kg		
		Elderly (64.3 ± 4.8)	PK STUDY	9.83 ± 2.55	3.84 ± 1.91	0.67 ± 0.15/kg	0.13 ± 0.06/kg		
Antifungal agents	Voriconazole (Cheng et al., 2020) (Dosage 6 mg/kg)	Adults (18–60)	PK STUDY	3.11 ± 2.13					
		Elderly (≥60)	PK STUDY	4.31 ± 3.03					
	Posaconazole Sansone-Parsons et al. (2007) (Dosage 400 mg)	Adults (18–25)	PK STUDY	2.607	31.6		33.1		26.740
		Elderly (≥65)	PK STUDY	3.374	41.8		26.7		35.444
	Isavuconazole Desai et al. (2019) (Dosage 372 mg)	Adults Female (22–45)	PPK				2.13		
		Elderly Female (66–84)	PPK				1.44		
	Terbinafine (Nedelman et al., 1997) (Dosage 250 mg)	Adults (18–40)	PK STUDY	1.631					9.500
		Elderly (60–80)	PK STUDY	1.963					12.489
Antiviral agents	Oseltamivir (Abe et al., 2006) (Dosage 75 mg)	Adults (20–35)	PK STUDY	0.061	1.45	1,350.44	652.75		0.119
		Elderly (≥80)	PK STUDY	0.093	1.64	845.52	358.40		0.214
	Bictegravir (Stader et al., 2021)	Adults (20–55)	PBPK	4.626 ± 3.208	18.9 ± 11.4	11.9 ± 3.8	0.627 ± 0.451		79.703 ± 76.782
		Elderly (55–85)	PBPK	4.971 ± 1.988	27.9 ± 15.0	10.8 ± 3.5	0.559 ± 0.451		89.423 ± 46.802
	Paxlovid	Adults (Chan et al., 2023) (18–86)	PPK			69.7	9.09		
		Elderly Qu et al. (2023) (74.8–85.0)	PPK			39.1	4.16		

Abbreviation: 1. C_max_: maximum concentration; 2. T_1/2_: half-life, V_d_: volume of distribution; 3. CL: clearance; 4. CL_r_: renal clearance; 5. AUC: serum concentration time curve; 6. PBPK: physiologically based pharmacokinetic; 7. PPK: population pharmacokinetics.

### 3.1 β-lactam antibiotics (penicillins, cephalosporins, atypical β-lactams, and β-lactam inhibitors)

β-lactam antibiotics, recognized as time-dependent agents, exhibit distinct PK characteristics in the elderly, primarily characterized by an augmented area under the serum concentration-time curve (AUC) and prolonged plasma half-life (T_1/2_). This phenomenon is evident across various penicillins, including penicillin, piperacillin, ampicillin, and amoxicillin. Notably, the AUC of amoxicillin in elderly subjects surpasses that of their younger counterparts (18,751 ± 3,903 h mg·L^−1^ vs. 37,542 ± 9,523 h mg·L^−1^), coupled with a lengthened T_1/2_ (1.43 ± 0.25 h vs. 1.75 ± 0.22 h) (Lee et al., 2017; Lee et al., 2020). Consistent findings emerge in other penicillin drugs (Yaoguo et al., 2001; Meyers et al., 1991). Similarly, cephalosporins demonstrate higher AUC and prolonged T_1/2_ in the elderly (Yaoguo et al., 2001; Mingji et al., 2007; Barbhaiya et al., 1992). For instance, a study on cefazolin reveals an elevated AUC in the elderly compared to adult subjects (380.71 ± 61.4 h mg·L^−1^ vs. 339.56 ± 79.9 h mg·L^−1^), along with a prolonged T_1/2_ (2.5 ± 0.4 h vs. 1.9 ± 0.3 h) (Yaoguo et al., 2001). Atypical β-lactams, including carbapenems and monocyclic β-lactams, exhibit analogous trends (Musson et al., 2004; Ljungberg and Nilsson-Ehle, 1992; Liu et al., 2013; Namkoong et al., 2014; Kim et al., 2018; Harada et al., 2013). For example, ertapenem’s AUC in the elderly surpasses that of the adult subjects (781.5 ± 95.9 h mg·L^−1^ vs. 572.1 ± 68.6 h mg·L^−1^), accompanied by a prolonged T_1/2_ (5.2 h vs. 3.8 ± 0.5 h) (Musson et al., 2004). Similarly, meropenem’s AUC in the elderly surpasses that of the adult subjects (58.3 ± 10.0 h mg·L-1 vs. 39.6 ± 6.8 h mg·L^−1^), accompanied by a prolonged T_1/2_ (1.27 h vs. 0.81 h) (Ljungberg and Nilsson-Ehle, 1992). Another population pharmacokinetics (PPK) study demonstrates that meropenem clearance rate (CL) is significantly lower in the elderly compared to CL reported in younger patients due to the reduced renal function (Usman et al., 2017). Aztreonam follows suit, with higher AUC in the elderly (469.01 ± 144.03 h mg·L^−1^ vs. 294.42 ± 64.08 h mg·L^−1^) and an extended T_1/2_ (3.10 ± 0.90 h vs. 1.80 ± 0.51 h) (Meyers et al., 1993). β-lactamase inhibitors, such as sulbactam, clavulanate, and avibactam, don’t directly inhibit bacteria but effectively prevent β-lactam antibiotics from hydrolysis by β-lactamase. In a PPK study on ceftazidime/avibactam (Li et al., 2019a), it has bee observed that avibactam’s AUC is higher in the elderly compared to younger subjects (156 h mg·L^−1^ vs. 131 h mg·L^−1^). In light of these findings, it is advisable for elderly patients to appropriately reduce dosage and closely monitor serum creatinine levels (Scr). This precaution is crucial as the risk of adverse drug reactions escalates with higher AUC and prolonged T_1/2_ (Berg and Hahn, 2001).

### 3.2 Aminoglycoside antibiotics

Aminoglycoside antibiotics operate as concentration-dependent agents, implying that a higher peak concentration intensifies their lethality against pathogenic bacteria and accelerates the killing speed. These antibiotics, characterized by low plasma protein binding, exhibit a propensity to accumulate in specific areas such as the renal cortex, inner ear, and perilymph fluid, thereby posing a heightened risk of nephrotoxicity and ototoxicity. Compounding this, the primary elimination route for these drugs is through glomerular filtration. In the elderly and patients with renal failure, the original forms of aminoglycosides can experience an extension of T_1/2_ by more than 20 to 30 times, elevating the likelihood of drug accumulation and subsequent poisoning in these populations (Fujita et al., 1985). The PK profile of aminoglycosides in the elderly predominantly manifests as an increased T_1/2_ and a diminished plasma CL. A PK study on subcutaneous tobramycin injection in both adults and elderly healthy volunteers reveals notable differences (Champoux et al., 1996). The T_1/2_ in the aged group is 3.53 ± 0.97 h compared to 2.73 ± 1.17 h in the adult group at an 80 mg dosage (*p* < 0.05). Additionally, CL in the elderly group significantly decreases to 5.28 ± 1.44 L h^−1^ from 6.9 ± 3.12 L h^−1^ in the adult group. Analogous outcomes are evident in studies involving gentamicin (Bos et al., 2019; Johnston et al., 2014) and amikacin (Yaoguo et al., 2001). Given these substantial PK changes, the elderly face a heightened risk of adverse drug reactions, necessitating strict dosage control based on PK parameters and therapeutic drug monitoring (TDM) during the administration of these medications (Hodiamont et al., 2022).

### 3.3 Macrolide antibiotics

With the exception of azithromycin, which falls under concentration-dependent antibiotics, all other macrolides are categorized as time-dependent antibiotics (Dorfman et al., 2008). Macrolide antibiotics have undergone three generations of development. The first generation, exemplified by erythromycin and dirithromycin, exhibits low bioavailability and is associated with common gastrointestinal reactions. The second generation, represented by azithromycin and clarithromycin, showcases improved bioavailability and a reduced incidence of side effects. The third generation, including telithromycin, demonstrates significantly enhanced bioavailability and antibacterial efficacy, with an absolute oral bioavailability of 57% in both adults and elderly individuals (Perret et al., 2002). Macrolide antibiotics are rapidly absorbed and widely distributed in extravascular tissues, with concentrations in tissues notably surpassing those in the bloodstream. Generally, in the elderly, age-related reductions in absorption and elimination lead to higher AUC and maximum concentration (C_max_) of macrolides compared to adult subjects. This trend is observed in telithromycin (Perret et al., 2002), dirithromycin (Varanese, 1993), and clarithromycin (Chu et al., 1992). However, a study on azithromycin reveals that while AUC is higher in the elderly compared to adult subjects (3.0 h mg·L^−1^ vs. 2.5 h mg·L^−1^), C_max_ shows only slight changes. Generally, adjusting dosing regimens based solely on age is unnecessary (Coates et al., 1991).

### 3.4 Tetracycline antibiotics

Tetracycline antibiotics, including tetracycline, minocycline, doxycycline, and tigecycline, are classified as time-dependent and are predominantly metabolized by the liver. It is crucial for patients with hepatic insufficiency to exercise caution and minimize the use of tetracyclines. Studies investigating the PK characteristics of minocycline reveal significant changes in multiple parameters when comparing adults and elderly groups. In a continuous intravenous infusion of 200 mg minocycline for the adult group and 100 mg for the elderly group, notable differences emerge (JB, 1983; Yamamoto et al., 1999). The C_max_ in the elderly group is twice that of the adult group (7.22 ± 3.53 mg L^−1^ vs. 3.5 mg L^−1^), accompanied by a substantial prolongation of T_1/2_ in the elderly group (25.0 ± 16.4 h vs. 15.7 h). Additionally, both V_d_ and CL are reduced in the elderly group. Similarly, a study on tigecycline has observed a larger C_max_ in elderly males compared to adult males (1.017 ± 0.112 mg L^−1^ vs. 0.861 ± 0.154 mg L^−1^), with a prolonged T_1/2_ in the adult group (22.3 ± 15.3 h vs. 19.0 ± 5.0 h) (Muralidharan et al., 2005). However, it’s noteworthy that these differences are attributed to variations in body size rather than age. Consequently, adjusting dosing regimens based solely on age appears unnecessary.

### 3.5 Quinolone antibiotics

Quinolone antibiotics, renowned for their concentration-dependent nature, have evolved into the fourth generation, with the first- and second-generation drugs seeing less use due to their lower blood concentrations. The third-generation quinolones, encompassing ofloxacin, levofloxacin, ciprofloxacin, norfloxacin, among others, and the fourth-generation quinolones like moxifloxacin and gatifloxacin, are widely embraced in clinical applications owing to their expansive antibacterial spectrum, potent antibacterial action, and minimal adverse reactions. Comparatively, the PK characteristics of third- and fourth-generation quinolones in the elderly differ from those in adult individuals, primarily marked by increased C_max_ and AUC (Nicolle, 1999). The elimination routes of quinolones predominantly involve a hepatorenal dual pathway, with renal elimination taking precedence. In clinical practice, the choice of quinolone is contingent upon the patient’s liver and kidney function. For instance, ofloxacin, predominantly eliminated through the kidneys, exhibits larger AUC in the elderly compared to the adults (83.3 ± 16.6 h mg L^−1^ vs. 21.5 ± 4.27 h mg L^−1^), along with a prolonged T_1/2_ (8.5 ± 1.2 h vs. 6.2 ± 0.9 h) (Molinaro et al., 1992). Therefore, the dose of ofloxacin for patients over 75 years old should be halved compared to adult patients. Studies on levofloxacin and sitafloxacin, which are mainly eliminated through the kidneys, yield similar results (Setiawan et al., 2022; Cojutti et al., 2017; Yaozh, 2023). On the other hand, ciprofloxacin, eliminated through both the liver and kidneys (Li et al., 2019b), does not undergo significant changes in PK parameters in the elderly (Ljungberg and Nilsson-Ehle, 1989), rendering dose adjustment generally unnecessary. Novel quinolones antibacterial agents, including garenoxacin (Van Wart et al., 2004; Ohsaki et al., 2010), nemonoxacin (Wu et al., 2015; Kang et al., 2019), delafloxacin (Hoover et al., 2016), and lascufloxacin (Totsuka et al., 2020), have not shown more pronounced adverse reactions. Therefore, it is recommended that the elderly use these drugs in the same manner as adult individuals.

### 3.6 Oxazolidinone agents

Oxazolidinone agents, a class of synthetically derived antibacterial agents that inhibit protein synthesis, are exemplified by oral linezolid, which boasts rapid and complete absorption with exceptionally high bioavailability. A PK study of linezolid indicates that its PK parameters do not significantly differ between the elderly and the adults, suggesting that age-based dose adjustment is unnecessary (Sisson et al., 2002). Tedizolid, a novel oxazolidinone antibiotic, exhibits a more favorable PK and safety profile compared to linezolid (Iqbal et al., 2022). An open-label phase I study on tedizolid reveals similar PK parameters across different age groups, affirming that dosage adjustments based on age are not warranted (Flanagan et al., 2018). Contezolid (MRX-I), another recently approved oxazolidinone agent in 2021 for treating complicated skin and soft tissue infections (cSSTIs) in China (Hoy, 2021), demonstrates potent activity against drug-resistant Gram-positive bacteria, with a higher safety profile than linezolid (Wu et al., 2020). A PPK study on contezolid suggests that age has no significant impact on the PK parameters, further supporting the conclusion that dosage adjustment based on age is unnecessary (Li et al., 2020).

### 3.7 Polypeptide antibiotics

Polypeptide antibiotics, encompassing polymyxins and glycopeptides, exhibit distinct PK characteristics. Polymyxins, including polymyxin B and polymyxin E (colistin), operate as concentration-dependent antibiotics. PPK studies (Manchandani et al., 2018; Wang et al., 2022a) on polymyxin B in adults and the elderly reveal a non-statistically significant difference in CL between adults and elderly subjects (2.5 ± 1.1 L h^−1^ vs. 1.98 ± 0.67 L h^−1^). Generally, dose adjustment is not deemed necessary for the elderly; however, TDM is advisable due to potential renal toxicity (Fiaccadori et al., 2016; Wang et al., 2021). Though there’s a dearth of studies on colistimethate sodium (CMS) pharmacokinetics in the elderly, initiating colistin at a low dose and closely monitoring renal function is recommended owing to its nephrotoxicity (Drugsatfda, 2023). On the other hand, glycopeptides, such as vancomycin, norvancomycin, teicoplanin, and travanin, are time-dependent antibiotics. Vancomycin and norvancomycin, primarily excreted by the kidneys, exhibit significantly longer T_1/2_ in the elderly compared to adults (Zhang et al., 2008; Guay et al., 1993). Therefore, it is advisable to reduce the dose of vancomycin and norvancomycin and closely monitor renal function in the elderly. Teicoplanin studies (Thompson et al., 1992; Rosina et al., 1988) suggest comparable CL and T_1/2_ in the elderly and adults; however, considerable variation in teicoplanin PK parameters related to renal function among the elderly necessitates dosage and administration interval adjustments based on Scr levels (Uhart et al., 2013). Travanin, with similar clearance in the elderly and the adults, does not require age-based dosage adjustments (Wong et al., 2008; Goldberg et al., 2010).

### 3.8 Antituberculosis agents

Many medications possess anti-tuberculosis properties, and this section concentrates on the pharmacokinetic profiles of first-line oral anti-tuberculosis agents, including isoniazid, rifampicin, ethambutol, and pyrazinamide. For isoniazid, regardless of whether individuals are fast or slow acetylators, there are no significant differences in pharmacokinetic parameters between adults and elderly subjects (Advenier et al., 1980). As a result, dosage adjustments for older adults are unnecessary. Early research has shown that when 450 mg of rifampicin is administered orally, there are no significant differences in rifampicin pharmacokinetics between older and middle-aged tuberculosis patients (Weng, 1990). Moreover, considering the increasing resistance to rifampicin, future studies should consider higher doses (Abulfathi et al., 2019). According to a clinical trial involving 100 Tanzanian tuberculosis patients, CL of ethambutol was found to decrease with age, while C_max_ and AUC increased with age (Denti et al., 2015). Additionally, a physiologically based pharmacokinetic (PBPK) study on ethambutol indicates that renal function is a significant covariate affecting interindividual variability in the apparent clearance of ethambutol (Hung et al., 2023). Therefore, it is recommended that elderly individuals use a lower dosage than adults individuals. Currently, only a PBPK study has demonstrated that CL of pyrazinamide correlated with creatinine clearance (*p* = 0.0349) and showed a trend toward smaller CL values with advancing age (*p* = 0.0778) (Peloquin et al., 1997). However, due to the lack of statistical significance in the latter finding, more studies are needed to further determine whether dose adjustments are necessary in elderly patients.

### 3.9 Antifungal agents

The pharmacokinetics of antifungal agents in the elderly predominantly focus on synthetic antifungal drugs such as triazoles and terbinafine. Elderly patients using antifungals primarily metabolized by the liver are more prone to developing liver diseases (Chien et al., 1997), necessitating cautious use of these drugs in this population and performance of TDM (Ashbee et al., 2014). A study on voriconazole reveals a significant increase in C_max_ in the elderly compared to adults (4.31 ± 3.03 mg L^−1^ vs. 3.11 ± 2.13 mg L^−1^) (Cheng et al., 2020). Due to reduced liver function in aged patients, lower dosages and longer dosing intervals for voriconazole are recommended (Li et al., 2023). On the other hand, a study on the pharmacokinetics of posaconazole oral suspension indicates that although C_max_, T_1/2_, and AUC in the elderly are higher than in adult individuals, the differences are non-significant, and dosage adjustment based on age is unnecessary (Sansone-Parsons et al., 2007). Similarly, a PPK study on isavuconazole has found that although CL is lower in the elderly than in adult individuals (1.44 L h^−1^ vs. 2.33 L h^−1^), the difference is not significant (Desai et al., 2019). Therefore, adjusting isavuconazole dosage solely based on age is deemed unnecessary. Moreover, despite significant differences in the PK parameters of terbinafine across different ages, the variations are not substantial enough to lead to greater toxicity. Hence, there is no need to alter terbinafine dosage based on age (Nedelman et al., 1997).

### 3.10 Antiviral agents

Oseltamivir, an anti-influenza drug, exhibits an AUC that is 80% higher in the elderly compared to adult individuals. However, it is well tolerated in very elderly individuals, and dose adjustment is typically not necessary in elderly patients (Abe et al., 2006). It’s crucial to use antiviral drugs judiciously in older individuals, particularly those living with HIV, due to a higher prevalence of multidrug therapy, potential inappropriate drug use, and drug-drug interactions (Guaraldi et al., 2018). Bictegravir, a novel integrase inhibitor recommended for most HIV patients by US and European guidelines, does not require dosage adjustment for age, except in older HIV patients with severe comorbidities, according to a recent PBPK study (Stader et al., 2021). Amidst the COVID-19 pandemic, Paxlovid has emerged as a promising drug for treating the virus (Amani and Amani, 2023). Recent PPK studies indicate that the CL of Paxlovid in adult individuals is approximately twice that in the elderly (9.09 L h^−1^ vs. 4.16 L h^−1^). In these studies, creatinine clearance is identified as a significant covariate affecting the CL of nirmatrelvir, suggesting that dosage adjustment for Paxlovid in elderly patients should be considered based on creatinine clearance (Chan et al., 2023; Qu et al., 2023).

## 4 Advances in pharmacokinetic research methods of antimicrobials agents in elderly population

Given their compromised immunity and increased susceptibility to underlying diseases, the elderly population faces heightened vulnerability to infectious diseases caused by diverse pathogens. Consequently, this demographic is more prone to encountering adverse drug events during clinical trials, thereby amplifying the intricacies of PK research in the elderly (Schlender et al., 2016). Therefore, to efficiently ascertain PK information and deliver precise dosage recommendations for elderly patients, it becomes imperative to explore alternative methods for clinical PK determination.

### 4.1 PBPK

PBPK model is a mathematical framework that uses NONMEM, PK-Sim and other software to perform PBPK modeling to simulates the absorption, distribution, metabolism, and excretion of drugs within various tissues and organs, based on knowledge from physiology, biochemistry, and anatomy (Nestorov, 2003; Sager et al., 2015). In recent years, thanks to modern modeling and simulation techniques, PBPK models have progressively emerged as a novel alternative for determining pharmacokinetics and pharmacodynamics in the elderly patient population (Cui et al., 2021).

Ciprofloxacin, a widely used fluoroquinolone anti-infective drug, has seen a surge in drug resistance, emphasizing the critical need to select effective doses based on its PK characteristics for rational clinical use. Schlender et al. (2018) have meticulously collected and screened clinical PK data for both oral and intravenous ciprofloxacin across all age groups. Subsequently, they have employed PK-Sim and the systems biology platform MoBi to construct PBPK models for both administration methods, validating these models in adults. Finally, they extend the ciprofloxacin PBPK model to encompass elderly and pediatric populations, comparing predicted exposure with clinical observations. Impressively, only 4.82% of intravenous doses and 12.13% of oral doses, on average, deviate from the range of the simulated data. Both the PBPK model for intravenous ciprofloxacin in adults and the PBPK model for oral ciprofloxacin in adults demonstrate reliability. When comparing predicted exposures for the elderly, children, and the entire life cycle with observed exposures, only 7.61% of intravenous dosing and 5.56% of oral dosing are anticipated to deviate beyond twice the average observed exposure. These findings highlight the ciprofloxacin PBPK model’s proficiency in accurately predicting exposure variations across different age groups and throughout the entire life cycle. The establishment and validation of this model are pivotal for optimizing dosage schemes and clinical trial designs for ciprofloxacin, particularly in special populations like elderly patients.

### 4.2 PPK

PPK integrates the PK model with a statistical model to quantitatively analyze the influencing factors of PK parameters in the patient population and the variability of PK when following a standard dose regimen. This analysis aids in proposing a more rational and effective alternative drug administration regimen (Sheiner and Ludden, 1992; Setiawan et al., 2023). Since 1998, the US Food and Drug Administration has permitted the use of population analysis in phase II and III clinical trials of new drugs to assess the PK/PD of special subjects (FDA, 1999). Notably, PPK studies of antibiotics in elderly patients have been on the rise.

Polymyxin B, as the last resort of multi-drug-resistant Gram-negative bacteria, pharmacokinetic data are currently scarce in the elderly population. Wang et al. (2022b) have established a PPK model using the Phoenix NLME software for critically ill elderly patients undergoing treatment with polymyxin B for multidrug-resistant Gram-negative infections. Nonlinear mixed-effect models and stepwise analysis methods are employed to explore the PK parameter covariate. Subsequently, based on the final PPK model, Monte Carlo simulations are conducted on three fixed-dose regimens (50 mg·12 h^−1^, 75 mg 12 h^−1^, and 100 mg 12 h^−1^) and weight-based regimens (1.25 mg kg·12 h^−1^ and 1.5 mg kg·12 h^−1^) to compare the efficacy and renal toxicity of each regimen. Results reveal that albumin is the primary covariate influencing PK parameters of polymyxin B in elderly patients. Simulation outcomes demonstrate that when MIC≤0.5 mg L^−1^, the probability of reaching the PK/PD target (ACU24 h/MIC >50) for all schemes is 90%. Fixed-dose regimens of 50 mg 12 h^−1^ and 75 mg 12 h^−1^ result in a relatively low likelihood of AUCss, 24 h > 100 mg h·L^−1^, at 53.9% and 66.7%, respectively. Therefore, these fixed-dose regimens of polymyxin B can achieve maximum efficacy while balancing renal toxicity. The PPK model of polymyxin B in elderly patients offers a safer and more effective administration scheme for critically ill elderly patients.

### 4.3 *In vitro* to *vivo* extrapolation (IVIVE)

IVIVE is a method utilized to forecast the rate at which drugs undergo metabolism in the human liver by leveraging *in vitro* drug metabolism measurements (Rowland and Pang, 2022). This process involves three key steps: initially measuring intrinsic clearance *in vitro*, subsequently establishing a correction equation for *in vitro* liver intrinsic clearance, and ultimately predicting liver clearance within the body (Sodhi and Benet, 2021). As IVIVE technology advances, its application scope has progressively expanded from animals to humans. Polasek et al. (2010) have employed IVIVE to assess the potential interaction between zolpidem and CYP3A-metabolized drugs, predicting its impact on the pharmacokinetics of this CYP3A-metabolized drug in humans. The anticipated results indicate that this interaction elevates the AUC of the drug in the elderly by 1–2 times. However, IVIVE does have limitations, including its tendency to exhibit poor accuracy in predicting *in vivo* data from *in vitro* measurements (Benet and Sodhi, 2022). Combining IVIVE with PBPK models can enhance prediction accuracy to a certain extent and assist in predicting drug clearance in the elderly (Polasek et al., 2013; Kim and Lee, 2022).

### 4.4 Machine learning (ML)

ML is a powerful method for analyzing and simulating large volumes of data, finding applications in diverse fields such as image recognition and language processing (Zhou et al., 2018; Popel et al., 2020). Its potential in the realm of PK analysis and prediction is considerable and can be harnessed for both adults and the elderly (Ota and Yamashita, 2022). Cheng et al. (2023) have employed 3 ML algorithms (random forest, gradient boosting, and extreme gradient boosting) to predict toxic plasma trough concentrations of Voriconazole (>5 μg mL^−1^). Results indicate that the prediction values from models established by these algorithms, including younger adult models, mixed models, and elderly models, are all meaningful. The study concludes that ML models can serve as reliable tools for predicting toxic concentration exposure of Voriconazole ML is broadly categorized into supervised and unsupervised learning. In comparison with PBPK and PPK models, ML possesses the ability to automatically re-establish mathematical relationships when data are updated, resulting in enhanced predictive performance (Gill et al., 2022). Furthermore, when compared with IVIVE and animal scaling (AS), ML exhibits economic efficiency and the capability to process extensive data and samples (Danishuddin et al., 2022). Moreover, innovative approaches involve combining ML with PBPK/PPK or integrating ML with IVIVE/AS. For instance, Kosugi and Hosea (2021) have explored a quantitative method for predicting the area under the plasma concentration-time curve after oral administration in rats by combining IVIVE with ML. The findings demonstrate that the amalgamation of ML and IVIVE significantly improves the ability to predict the area under the plasma concentration-time curve after oral administration.

## 5 Conclusion

Given the expanding elderly population and the critical need for precise anti-infection treatment tailored to this demographic, coupled with the limited research on numerous promising antimicrobial agents in the elderly, there is an urgent call to comprehensively delineate the PK characteristics of these agents in elderly patients and formulate rational administration schemes. Furthermore, the current PK study methods for the elderly exhibit certain limitations. Therefore, it is imperative to not only optimize existing PK research methods but also explore innovative approaches to address these limitations and enhance our understanding of drug dynamics in the elderly.
